# [Expression of Concern] Multiple roles of SOCS proteins: Differential expression of SOCS1 and SOCS3 in atherosclerosis

**DOI:** 10.3892/ijmm.2026.6006

**Published:** 2026-09-30

**Authors:** Xiao Liang, Ming He, Tao Chen, Yan Liu, Yu-Ling Tian, Yu-Liang Wu, Yan Zhao, Yan Shen, Zu-Yi Yuan

Int J Mol Med 31: 1066-1074, 2013; DOI: 10.3892/ijmm.2013.1323

Following the publication of the above paper, it has been drawn to the Editor's attention by a concerned reader that, for the immunohistochemical images shown in Fig. 3 on p. 1070, the 'SOCS3/HD/16W' and 'SOCS3/CD/28W' images were apparently the same, albeit with differently coloured shading, suggesting that this figure had been assembled incorrectly. In addition, upon analyzing the data in this paper independently in the Editorial Office, it came to light that one of the histological images featured in Fig. 2A (the 'Oil Red O/12W' image) apparently reappeared in a paper written by different authors at different research institutes in the journal *Redox Biology* several years after the publication of this article, albeit the image in that paper did not feature the same level of clarity.

The authors have been contacted by the Editorial Office to offer an explanation for the apparently incorrect assembly of the data in Fig. 3, and we are awaiting their response. Owing to the fact that the Editorial Office has been made aware of potential issues surrounding the scientific integrity of this paper, we are issuing an Expression of Concern to notify readers of this potential problem while the Editorial Office conitnues to investigate this matter further.

